# The Fluid-Dynamics of Endo Vascular Aneurysm Sealing (EVAS) System failure

**DOI:** 10.1007/s13239-021-00520-3

**Published:** 2021-02-09

**Authors:** F. Battista, R. Ficarelli, A. Perrotta, P. Gualtieri, C. M. Casciola, G. P. Romano, M. Taurino

**Affiliations:** 1 Department of Mechanical and Aerospace Engineering, Sapienza University of Roma, Roma, Italy; 2 Department of Clinical and Molecular Medicine, Sapienza University of Roma, Roma, Italy

**Keywords:** Abdominal aortic aneurysm, Endovascular aneurysm sealing, Endobags, Computational fluid dynamics

## Abstract

**Purpose:**

The main objective of this work is to investigate hemodynamics phenomena occurring in EVAS (Endo Vascular Aneurysm Sealing), to understand if and how they could lead to type 1a endoleaks and following re-intervention. To this aim, methods based on computational fluid mechanics are implemented as a tool for checking the behavior of a specific EVAS configuration, starting from the post-operative conditions. Pressure and velocity fields are detailed and compared, for two configurations of the Nellix, one as attained after correct implantation and the other in pathological conditions, as a consequence of migration or dislocation of endobags.

**Methods:**

The computational fluid dynamics (CFD) approach is used to simulate the behavior of blood within a segment of the aorta, before and after the abdominal bifurcation. The adopted procedure allows reconstructing the detailed vascular geometry from high-resolution computerized tomography (CT scan) and generating the mesh on which the equations of fluid mechanics are discretized and solved, in order to derive pressure and velocity field during heartbeats.

**Results:**

The main results are obtained in terms of local velocity fields and wall pressures. Within the endobags, velocities are usually quite regular during the whole cardiac cycle for the post-implanted condition, whereas they are more irregular for the migrated case. The largest differences among the two cases are observed in the shape and location of the recirculation region in the rear part of the aorta and the region between the endobags, with the formation of a gap due to the migration of one or both of the two. In this gap, the pressure fields are highly different among the two conditions, showing pressure peaks and pressure gradients at least four times larger for the migrated case in comparison to the post-implanted condition.

**Conclusions:**

In this paper, the migration of one or both endobags is supposed to be related to the existing differential pressures acting in the gap formed between the two, which could go on pushing the two branches one away from the other, thus causing aneurysm re-activation and endoleaks. Regions of flow recirculation and low-pressure drops are revealed only in case of endobag migration and in presence of an aneurysm. These regions are supposed to lead to possible plaque formation and atherosclerosis.

## Introduction and Purposes

There is a great debate on the effective reliability of the EVAS technique in the wide offer of endografts to treat an Abdominal Aortic Aneurysm (AAA), especially in comparison to fixed non-sealing devices.^6^ Both sealing and non-sealing systems require using endo-devices, placed upstream of the aortic abdominal bifurcation, inserted through the femoral artery, and then guided up to the aneurysm neck.^10^ The main advantages of such solutions reside in avoiding a local invasive operative procedure, in ensuring almost complete separation of the aneurysm from the local blood flow circulation, and in a rather simple positioning procedure.

Nevertheless, almost all configurations of endograft used to treat AAA, retain a significant risk of endoleak, which is a complication, due to antegrade or retrograde reperfusion of the sac, requiring a different kind of re-treatment. This occurrence has been widely described and classified and the onset of endoleak within one year from the surgery has been reported to be around 5% for EVAS, in comparison to more than 10% for other endovascular repair systems.^4^,^5^,^15^

There are five different types of endoleaks, classified on the basis of features causing the back-flow into the aneurysm sac.^13^ Such endoleaks are coupled to high local pressures and consequently require re-intervention after implantation, to prevent vascular rupture (around 1% of the total number of implantations after one year and rising to more than 2% after the third year).

Specifically, among the different classifications of endoleaks after EVAS, Type Is1 endoleak is defined as the appearance of contrast between the endobag and the wall of the proximal neck, but not reaching the aneurysm sac itself. In Type Is2 and Is3, the aneurysm sac is reached, in the last case with the appearance of contrast or fresh thrombus between the endobags. Lastly, in Type Is4, the presence of sac pressurisation is observed.

In this work, the attention is focused on type Is3, i.e. in the case of EVAS involving unexpected flowing blood in between the endobags and the native vascular system. As reported, these conditions lead to relapses and even considering that many of those are due to specific anatomic and pathological complications (short length of the neck, large bifurcation angles, large lumen, large aneurysm diameter), other reasons for such post-operative consequences should be investigated with care. From this point of view, there are suggestions for the migration of endo-devices.^16^ This condition could be considered a direct cause of the aortic rupture and could be also a concurrent cause for endoleaks.

Hence, careful monitoring of endoleak absolute and relative positioning is required, as this process cannot be predicted. This uncertainty has relevant consequences about the final geometrical configuration of endo-devices and sealing material at the aortic neck. The long-term operative success could be affected by the interaction of the specific EVAS configuration with the blood flow and by the consequent hemodynamic effects. The resulting changes in the duration and degeneration of tissues and materials must be continuously and carefully monitored with related high increasing costs and multiple re-intervention to rearrange the endo-devices.^17^,^18^

**Figure 1:**
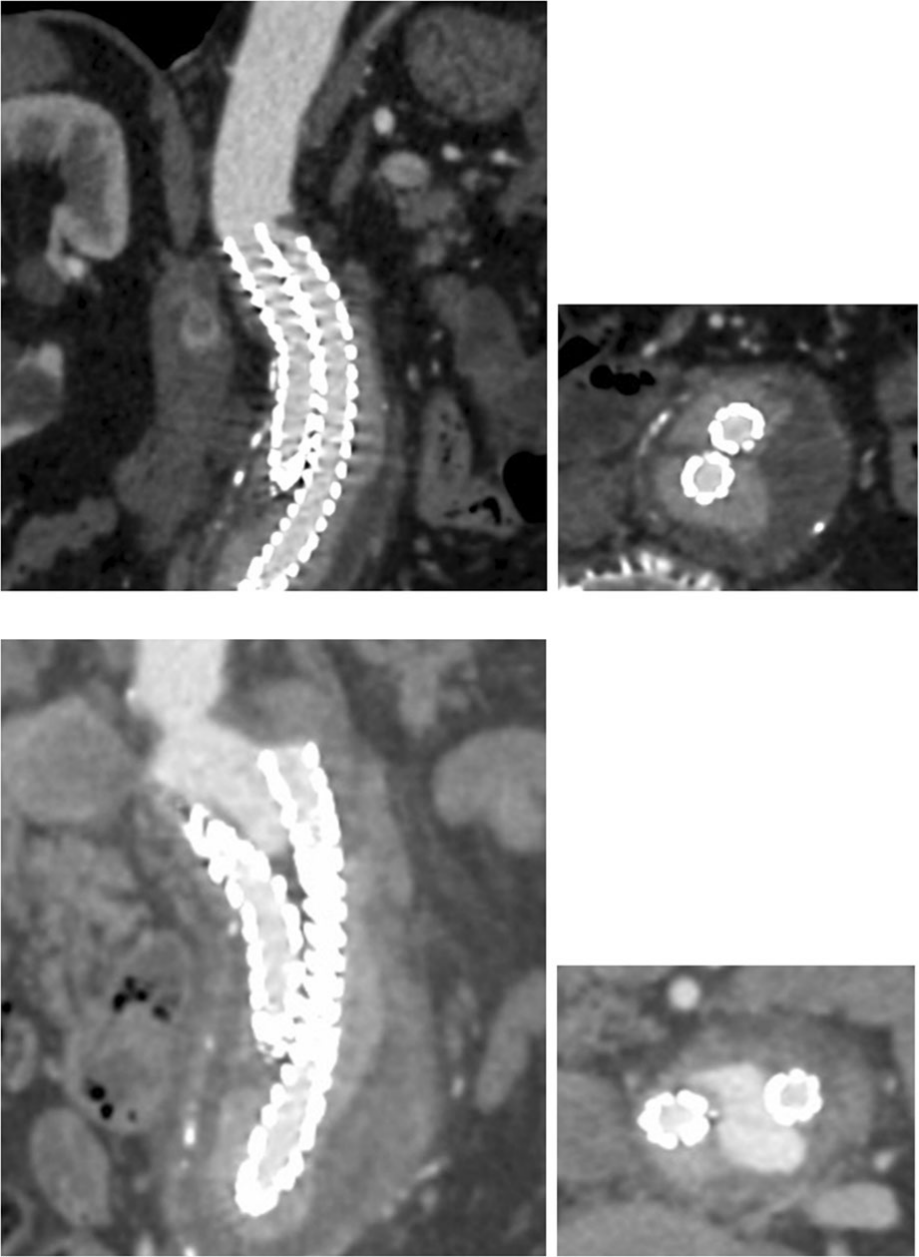
Lateral (on the left) and cross (on the right) views of endobag configuration after implantation (at the top) and after revealing post-operative migration (at the bottom) as obtained with CT scan and Contrast Imaging, as acquired at Sapienza University.

Among the others, the Nellix® EVAS system (Endologix Inc, Irvine, CA, USA) consists of locally expandable stents, coupled with two polymer bags (endobags), positioned just below the renal arteries, to completely separate the aneurysm from the flowing blood. The entire aneurysmal sac is filled by the polymer enclosed in the endobags, thus avoiding the back-flow through the lumbar or inferior mesenteric artery. As remarked, the final exact position and geometrical configuration attained by the polymer at the end of implantation procedures have a large degree of unpredictability. This is also coupled with unexpected movements and migration of endobags in time, occurring after the intervention, and could contribute to the requirement of successive re-intervention. Indeed, the final configuration of the two endobags could also strongly affect the durability and degeneration of the entire system. An example of the migration phenomenon is reported in Fig. 1, obtained with Computerized Tomography (CT scan) and Contrast Imaging, in which the after implantation and migrated configurations of the Nellix system are presented and compared.

As a consequence of all previous arguments, it would be extremely interesting to investigate and detail the fluid-dynamics of Nellix EVAS systems and to compare them with other known conditions, pathological or not.

In this work, the computational fluid dynamics (CFD) approach is used to investigate two configurations of the Nellix EVAS endobags, one after successful implantation of the devices and the other as revealed in pathological conditions due to migration of endobags. The work aims to investigate the fluid mechanics phenomena taking place in the two configurations and to compare them in terms of local pressure and velocity fields, to understand which specific phenomenology, present in the second and not in the first case, could lead to endoleaks and need for re-intervention. Both configurations are compared with reference conditions, like those of a healthy patient and pathological conditions with an extended aneurysm.

## Methods and Materials

High-resolution images derived from CT scans, as those reported in Fig. 1, allow the generation of three-dimensional and cross-sectional detailed views of a patient-specific aortic anatomy. Images are carefully analyzed to identify and isolate the specific aorta section under investigation. In this phase is important to include in the considered domain all vessels and branches which require the assignment of boundary conditions.

Four different patients with representative conditions were analyzed, the first one with an optimal clinical and hemodynamic condition, two years after the operation, the second one after a failed endovascular exclusion, which needs a surgical conversion, the third and fourth with rather standard healthy and aneurysm conditions, used as a reference. All patients gave informed consent for investigational use of their images.

**Figure 2:**
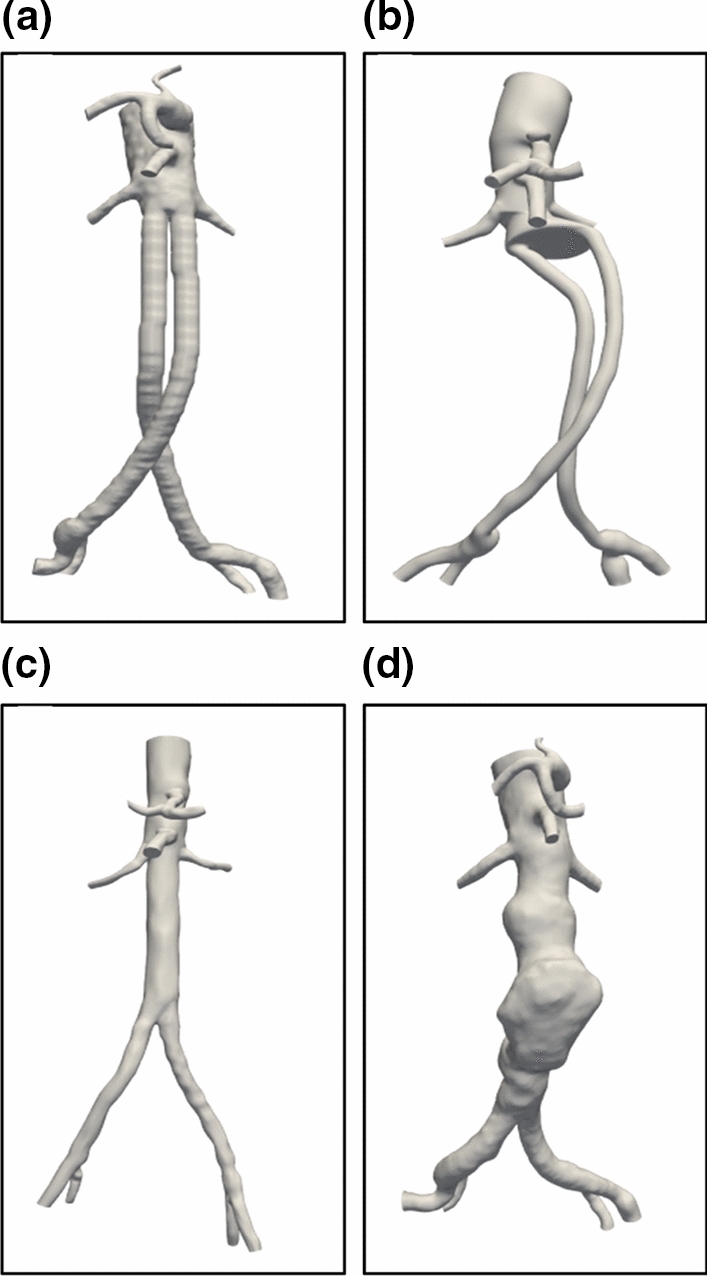
Front views of the four specific geometry investigated by CFD in this paper. Nellix EVAS immediately after intervention, configuration A (top left), Nellix EVAS after lateral migration, configuration B (top right), healthy patient, configuration C (bottom left) and presence of aneurysm, configuration D (bottom right).

Starting from such CT scan images, the SimVascular software^8^,^16^ allows restoring an approximation of the real vessel geometry and to discretize the selected computational domain. Specifically, from acquired images, the axis of each main vessel is identified and discretized steps, so that the vessel cross-section is defined (2D segmentation). Then, all cross-sections are linked together in order to define the overall structure of each vessel and the process is repeated for all vascular branches. The final result is a fully three-dimensional model of the test section, which represents the computational domain, like shown in Fig. 2. The model is tested with a trial-and-error procedure, in order to verify accuracy, uniformity, and continuity of the segmentation process, this step requiring great care and being also time-consuming.

The blood is simulated using the Newtonian model and all vessel walls are considered as rigid. The fluid flow is solved using the incompressible Navier-Stokes equations without any turbulence model, being the Reynolds number sufficiently small. Indeed, during a heartbeat, the bulk Reynolds number varies from $Re_{bulk} \simeq 200$ to $Re_{bulk} \simeq 1600$, corresponding to a maximum friction Reynolds number equal to $Re_{\tau } = u_{\tau } \rho R/\mu \simeq 100$, where $u_{\tau } = \sqrt {\tau_{w} /\rho }$ is the friction velocity, τ_w_ is the wall shear-stress, $\rho$ is the blood density, and $\mu$ is the blood kinematic viscosity. Then the estimated viscous length scale is equal to $y = \mu /\rho u_{\tau } \simeq 0.01 R \simeq 0.015\,{\text{cm}}$, which is comparable to the near-wall typical grid spacing $\Delta = 0.006R = 0.01\,{\text{cm}}$ (in viscous units $\Delta^{ + } = \rho u_{\tau } \Delta /\mu = 0.6$). Therefore, the resolution of the final computational grid is suitable to resolve the smallest fluid dynamics scale involved.

The computational grid is generated by using TetGen,^7^ an open-source software, based on the three-dimensional Delaunay triangulation. The grid spacing is non-uniform, being reduced at locations where a higher resolution is required, for example close to boundaries (vessel walls) and bifurcations. The CFD solver is embedded in SimVascular.

In particular, the Navier–Stokes equations in a three-dimensional arbitrary domain are discretized by means of a Finite Element Method (FEM) PHASTA (Parallel Hierarchical Adaptive Stabilized Transient Analysis) algorithm. It allows obtaining solutions in arbitrary domains,^20^ also ensuring good matching among the required computing accuracy and geometrical flexibility. The code is able to run on multiple cores, using the Message Passing Interface (MPI) paradigm, with well-documented scalability features on large clusters.^22^

Dealing with boundary conditions, the following are implemented and are constant for all tested cases:

- on the vessel walls, the velocity components are considered null and the wall is considered as rigid, without deformation;
- at the inlet section, the velocity profile is assigned, by enforcing the Womersley profile^21^ for laminar pulsatile flow, the flow rate and pulse period corresponding to the physiological condition (70 mL per beat, 70 mL/s, and 1 s, respectively);
- at the outlet sections, the systemic contributions are simulated by the so-called RCR condition (resistance-capacity-resistance), through a circuit including a resistance followed by a resistance-capacity pair in parallel. This has been evaluated as the most proper to simulate the systemic contribution, the specific model parameters being evaluated by following the procedure described in Ref. 19.

**Figure 3:**
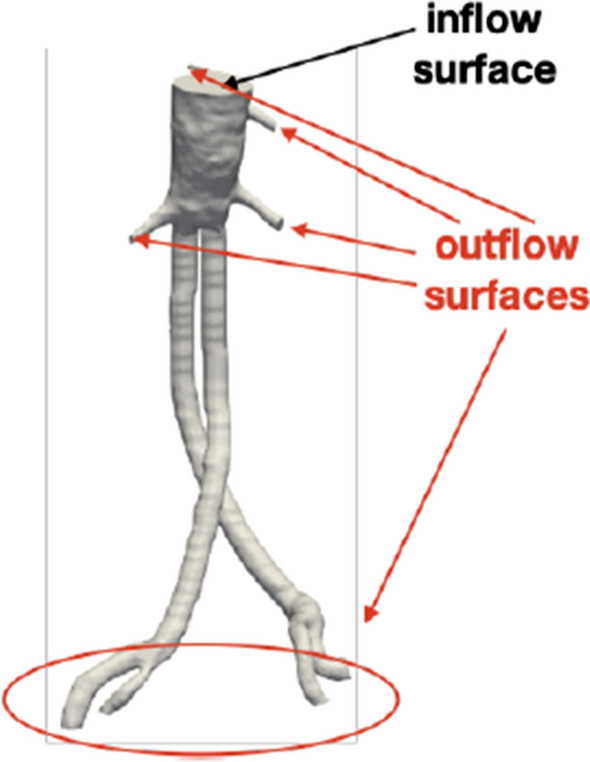
The specific tested geometry and details of the inlet (in black) and outlet (in red) surfaces used for imposing boundary conditions. The example is for the configuration A of the Nellix EVAS system.

The main inlet and outlet surfaces are summarized in Fig. 3. The total number of cells for each configuration is around 3.5 million.

It is worth highlighting that boundary conditions (as for example the specified inlet velocity profile) have a deep impact on the overall flow topology and specifically on wall-shear stresses, see Armour *et al*.^2^ for details. However, the main objective of the present work is to detail and compare the different tested geometries, rather than to investigate specific patient physiological parameters.

The last step is to select the temporal increment and the total duration of the simulation. The transient before reaching stable periodic conditions is equal to four heart cycles, except for case B which requires three more cycles. Then, for the other four cycles, all fluid mechanics quantities are registered, corresponding to a total computing time of 24 hours on 8 cores. Data analysis is performed using the open-source data visualization software Paraview.^1^,^3^

Four different geometries are considered, with two test cases involving Nellix EVAS. In the first (hereafter indicated as configuration A), the two endobags are correctly positioned, without any relevant spacing between them, as immediately after the intervention. In the second configuration (hereafter indicated as B), one of the two endobags migrated laterally, thus creating a significant gap between the two stents. The two conditions are reported at the top of Fig. 2, where front views are provided.

As can be noticed, the whole geometrical configuration of case B is highly different from case A. Specifically, on the right-hand side of the figure at the top, the large gap between the two endobags is clearly observed in comparison to the post-implantation condition reported at the top left.

Among the two reference cases considered, one involves a healthy patient (configuration C) and the other a vessel deformed by the presence of an aneurysm (configuration D), as also presented in Fig. 2 at the bottom. The reference configurations are included in the present analysis in order to have a basis for comparison and discussion, to highlight effects on blood pressure and velocity fields.

## Results

**Figure 4:**
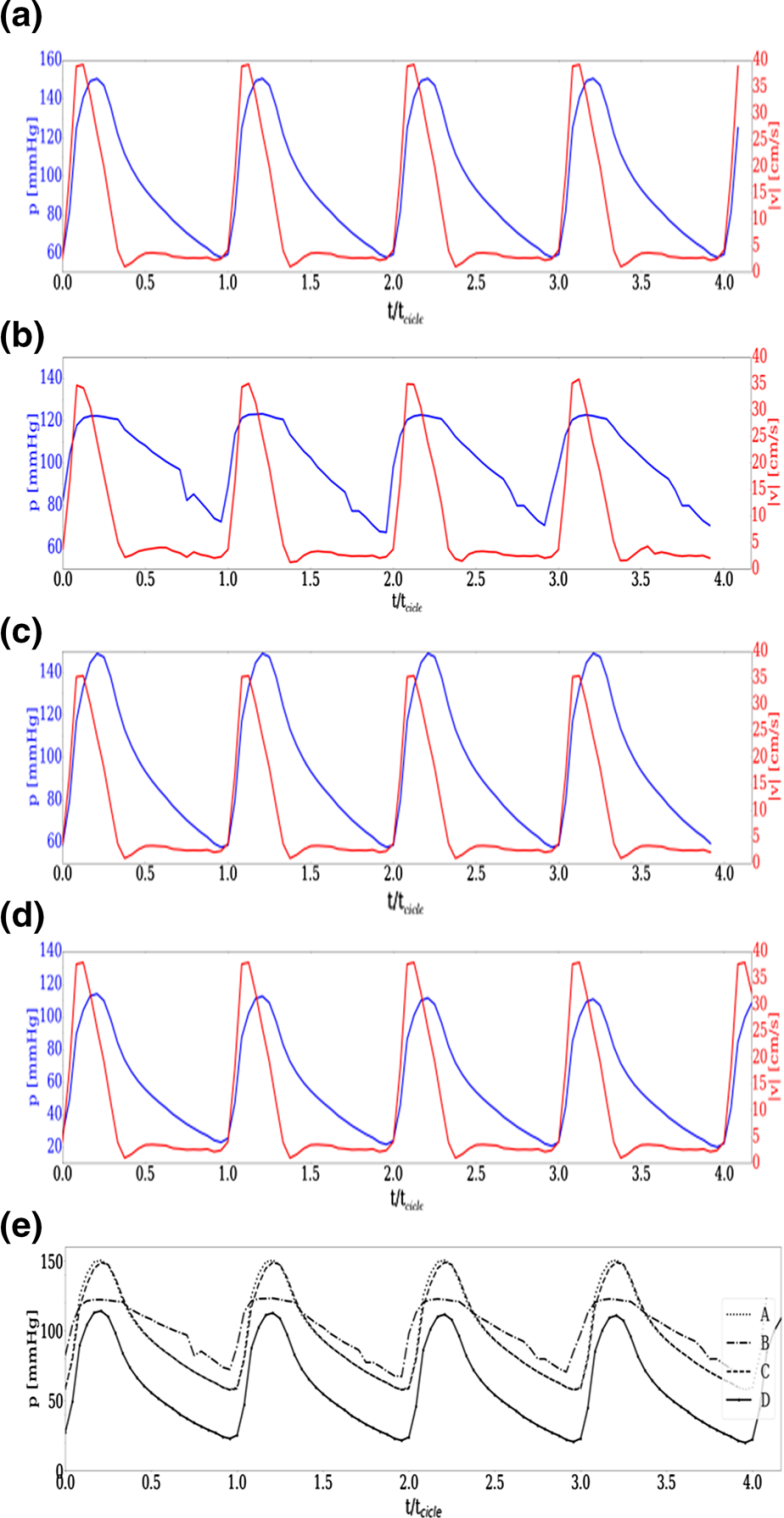
Temporal profiles of bulk velocity (computed from the flow rate) and of the mean pressure computed at a section near the inlet during four heartbeats. Panels from (a) to (d): velocity (in red, right hand scale) and pressure (in blue, left hand scale) variation during four heartbeats as derived at the inlet section. Panel (a): configuration A; panel (b): configuration B; panel (c): configuration C; panel (d); configuration D; panel (e): pressure temporal profiles for all four configurations.

In Fig. 4, panels from (a) to (d) report the variation of bulk velocity (in red, as obtained by the flow rate) and of mean pressure (in blue), close to the inlet section during the four heartbeats after transients. Since the volumetric flow rate is imposed at the inlet, the bulk velocity temporal profile is very similar in all considered conditions (variations are not larger than 15%, due to the different details of the geometrical configurations). As already reported, the volumetric flow rate is kept constant among all tested cases, in order to point out specific features due to geometrical conditions at the bifurcation. Thus, as a consequence of the established hemodynamic conditions, the physiological values in the tested cases are different. In any case, the aim of the present work is not to analyze specific physiological values, but to isolate the effect of the bifurcation geometry on fluid dynamics.

Panel (e) of Fig. 4 compares in the same plot the mean pressure variation for all four cases. Indeed, pressure variations are expected to strongly depend on the specific geometrical and flow configuration, so that also the downstream flow behavior could influence the upstream conditions. Considering that pressure boundary conditions are not imposed at the inlet (as reported in the previous paragraph), it is expected that some peculiar condition at aorta bifurcation would be back-reflected upstream, near the inlet section. Hence, in this sense, the pressure signal could carry footprints of pathological behavior of the blood flow field. Indeed, this is just the case of configurations B and D, where a pathological behavior is evident, whereas in cases A and C (which have similar pressure signals) pressure values are almost physiological. Specifically, in case B the temporal profile shows a large plateau of high pressure with abrupt variations, whereas in case D, the behavior in time seems regular, but pressure values appear to be very small.

The direct comparison of cases A and B, points out that the peak pressure in B is reduced by about 20% while the minimum pressure is increased, resulting in an average pressure much higher (being close to the maximum for almost 40% of the heart cycle, whereas in case A this happens for no more than 20%).

**Figure 5:**
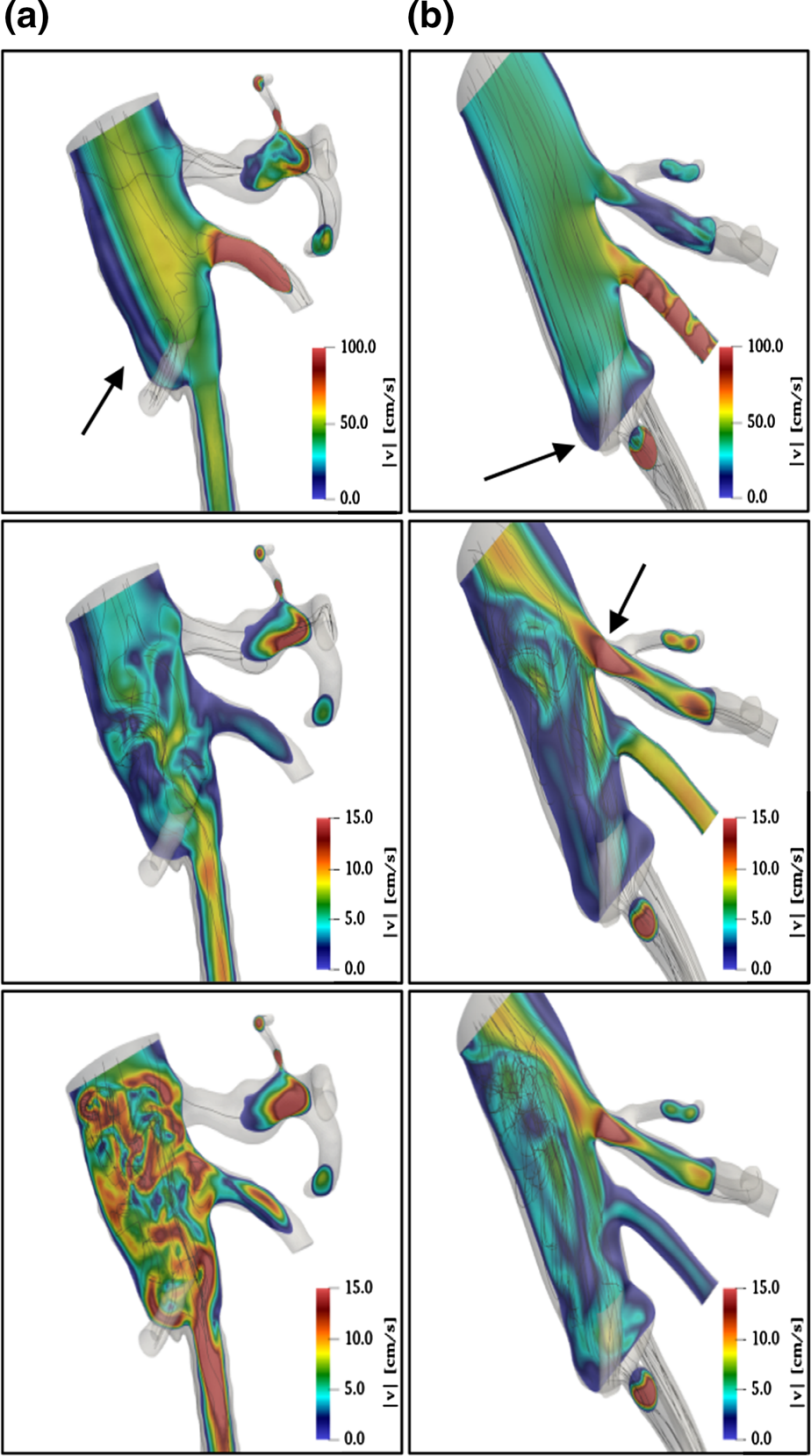
Lateral views of the velocity magnitude (in colors) and of streamlines (black lines) in geometrical configurations, (a) (on the left) and (b) (on the right), at three instants during the heartbeat: systolic peak (first row), diastolic minimum (second row) and at mean arterial pressure (third row). Main flow from top to bottom. Note the different colour scales.

Detailed lateral views of the velocity flow fields are reported in Fig. 5. Specifically, velocity magnitude and streamlines are reported at three different instants during the heart cycle, i.e. at the pressure systolic peak (*p* = *p*_sys_, taking place around *t*/*T* ≈ 0.2, where *T* is the heartbeat period) on the first row, at the diastolic pressure (*p* = *p*_dia_, taking place around *t*/*T* ≈ 1) on the second row, and at the mean arterial pressure (MAP) on the third row

$$ p_{MAP} = \frac{{p_{dia} + \, \left( {p_{sys} - p_{dia} } \right)}}{3}. $$

At the systolic peak, for configuration A (top left part of Fig. 5), the velocity magnitude is around 70 cm/s in the aorta and even larger in the kidney branches. The streamlines are quite regular and aligned with the axis of each branch, without major recirculation regions, except for the rear region of the aorta, before the bifurcation, where a blue region of low velocity is observed.

This is mostly originated by the stagnation region close to a small cavity, positioned laterally at the EVAS (in the figure, this is pointed out by the black arrow), allowing the flow to recirculate. This effect is only marginal and the flow in the endobags remains quite regular and almost undisturbed.

As already pointed out, the geometry of configuration B (top right part of the figure) substantially differs from A as a result of the endobags relative displacement. The maximum velocity is slightly smaller and most importantly the recirculation region is strongly modified, as shown by the low-velocity regions in blue colors.

Indeed, the recirculation located in the rear part of the aorta is significantly reduced in comparison to configuration A, and an extended new blue region forms in the large gap between endobags, as indicated by the arrow. In addition, in comparison to case A, the streamlines in the endobag are quite irregular, featuring oscillations downstream of the inlet. This seems to be a consequence of the new branch position relative to the aorta, as due to endobag migration. When reaching the diastolic pressure (second row in Fig. 5) and MAP (third row), the velocity magnitude is smaller, the typical velocity in the endobags being around (15–20) cm/s. Nevertheless, in the endobags, the flow field is still regular in comparison to the aorta, where the fluid is highly recirculating and three-dimensional, as pointed out by considering streamlined configurations.

Large regions of backflow are also observed, especially before the bifurcations into the lateral branches and into the endobags. In any case, the flow configuration is more regular for case B than for case A. Especially in case B, the rather large recirculation region in the rear part of the aorta partially diverts the flow from the endobags towards the kidney branches, as indicated by the arrow. This situation is also observed at MAP.

**Figure 6:**
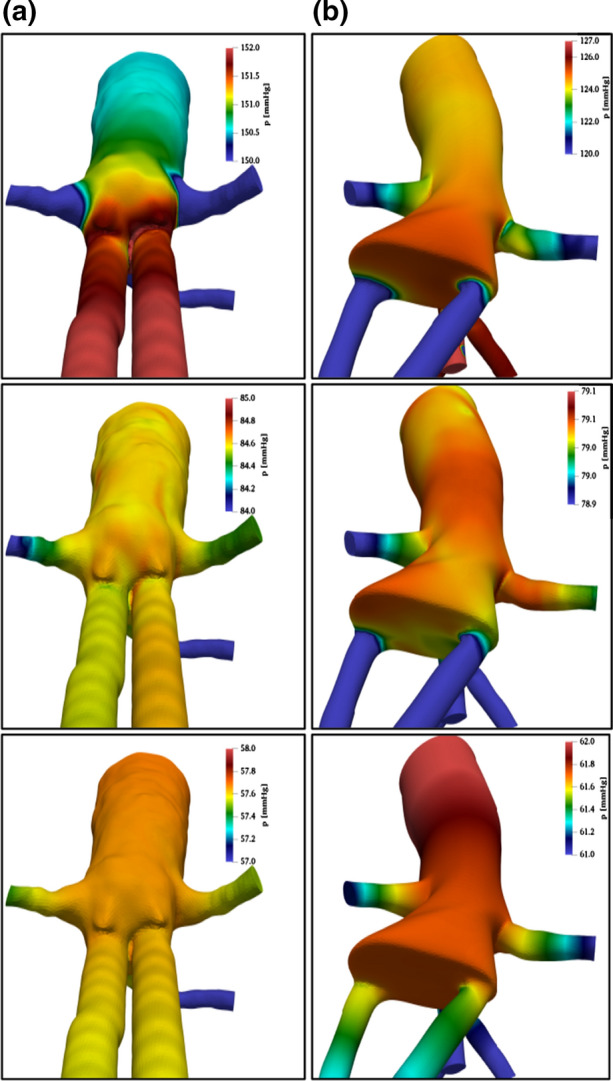
Rear views of wall pressure field (in colors) in geometrical configurations, (a) (on the left) and (b) (on the right), at three instants during the heartbeat: systolic peak (first row), diastolic minimum (second row) and at mean arterial pressure (third row). Main flow from top to bottom.

Wall pressure fields, in the same conditions of Fig. 5, are presented in Fig. 6 from a rearview, which highlights differences induced by endobag displacement.

Pressure fields are especially relevant in this context, since pressure forces may induce endobag migration and relocation.

For both cases, the maximum pressure is obtained at the systolic peak (first row), in correspondence to the aortic abdominal bifurcation.

However, there are important differences. While in the post-implanted configuration (A) the region of maximum pressure extends from the aortic root inside the endobags, for the migrated configuration (B), the pressure peak is concentrated in the gap between the two.

Moreover, in case A, wall pressure is spatially more uniform, with variations limited to 1%, in comparison with B where they increase to more than 5%. The mechanical implication is that the differential pressures acting at the endobag inlet sections may tend to separate the two branches. This behavior persists during the heart cycle, even if with reduced pressure values.

Since in case B high pressures are concentrated just at the gap between the endobags, the resulting loading effects are amplified during the whole cycle.

**Figure 7:**
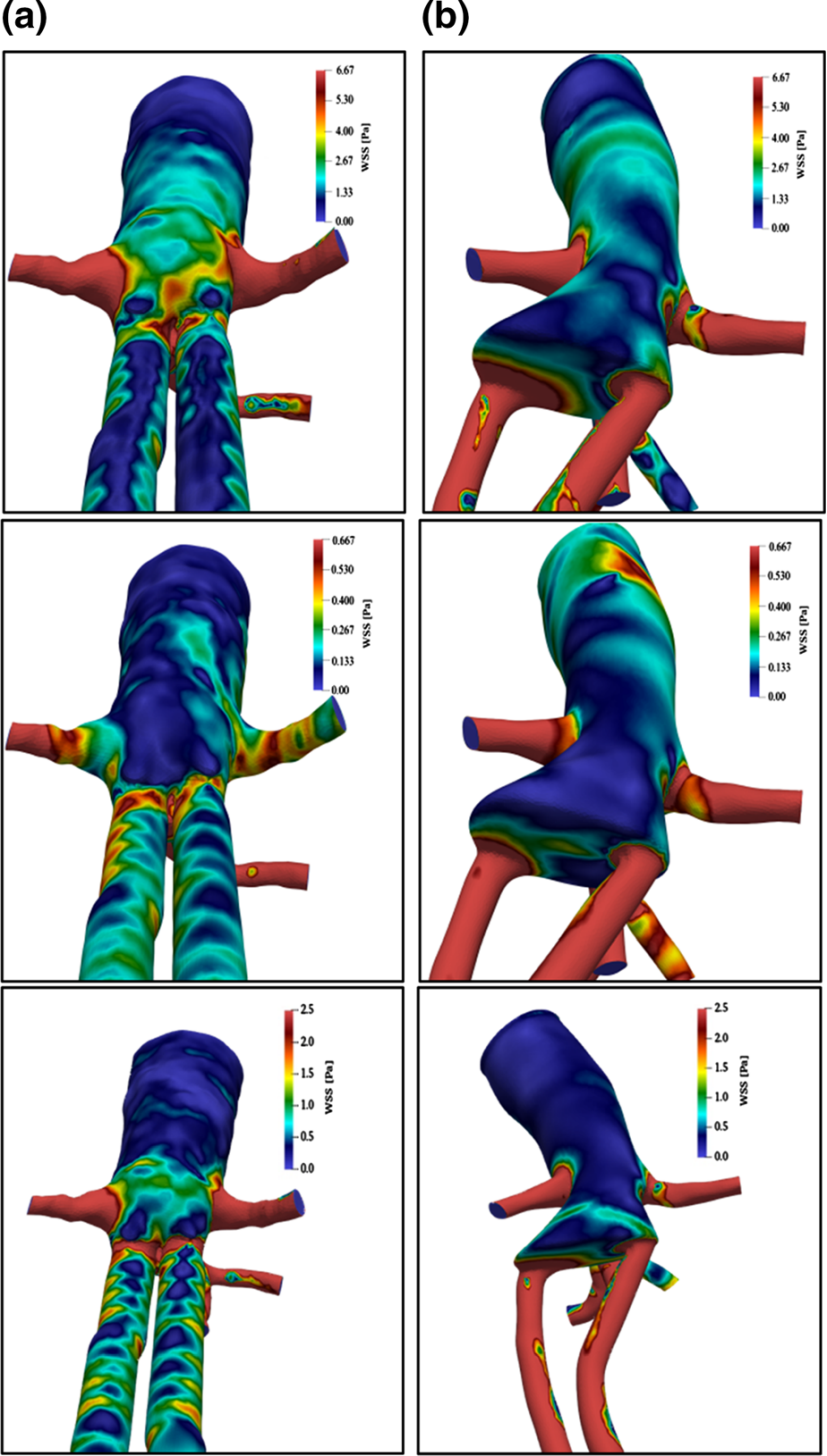
Rear views of wall shear stress (WSS) (in colors) in geometrical configurations, (a) (on the left) and (b) (on the right), at two instants during the heartbeat: systolic peak (first row), and diastolic minimum (second row). The time averaged value is reported in the third row. Main flow from top to bottom.

A further quantity to be addressed is the wall shear stress (WSS), as shown in Fig. 7. In this case, the attention is focused on the behavior at the systolic peak, at the diastolic minimum, and also on time-averaged values. Data obtained at MAP are no longer considered since the conclusions reached at this pressure value are the same as those obtained at the minimum.

As expected, in terms of magnitude, the contribution of the shear stress to the forces acting on the walls is almost irrelevant (less than 1/1000 of the pressure counterpart), confirming that mechanically it hardly contributes to endobags migration and relocation. The shear stress is however worth investigating since its anomalous values are well known to affect the chemical and physical properties of both endothelium and blood. Indeed, the WSS distribution indicates significant dissimilarities between the two cases, especially in the gap region between the endobags and at the walls.

The blue color in the pressure peak figure corresponds to regions where WSS < 1.3 Pa, which is a critical value for the possible formation of thrombus and atherosclerosis development.^9^ It is clearly observed that in all conditions (but especially for time-averaged values), for case B the blue regions have larger extensions, definitely moving towards the aorta bifurcation. Considering that also the changes in WSS intensity correlate with atherosclerosis, the gap region between the endobags in case B calls for attention, whereas in case A critical values are mostly concentrated on vessel walls.

## Comparisons and Discussion

In order to explain the failure of an EVAS system implantation, a possible strategy is employing advanced computational fluid dynamics (CFD) algorithms and codes, in order to check and detail the fluid-mechanics in post-operative and modified pathological conditions.

In the context of numerical simulation, a number of studies have been devoted to the investigation of the hemodynamics after the endovascular aneurysm repair. Casciaro *et al*.^5^ quantified the hemodynamic changes following EVAS, by comparing them with those revealed in EVAR, assuming steady conditions, pure Newtonian fluid with constant viscosity, rigid vessel walls, and outflow boundary conditions without any effect of systemic contributions. The authors observed higher values of velocity gradients and wall shear stresses in EVAS when compared to EVAR.

Raptis *et al.*^11^ compared the effect of AFX Endovascular System (Endologix, Inc., Irvine, CA, USA) and Nellix on the blood dynamics after implantation. The blood is modeled as pure Newtonian fluid and no-slip conditions are enforced at the rigid vessel walls. They observed that blood flow restoration is achieved with AFX, although a low-pressure drop is measured in limbs and secondary flow in the upper part of the endograft. The Nellix is found hemodynamically efficient, although a significant decrease of vorticity transport at the inlet of the endograft is observed. This study was extended by the same authors^12^ in four post-EVAR stent graft systems, characterized by a different design, material, and type of fixation, observing reliable differences, especially in the bifurcation region. The Nellix system seemed to produce more favorable conditions, with respect to the other stent-grafts, since it induces larger velocity and wall shear stresses and lower vorticity transport in the bifurcation region.

Tasso *et al.*^14^ studied the effect of two different commercial endovascular devices on local blood dynamics. The fluid is considered Newtonian and the vessel walls rigid. The authors investigated the modification of the geometry and fluid dynamics of the two devices and the forces acting on them, which can be responsible for the possible device migration. The authors highlight that the treated patients present higher torsion, curvature, and area variation rate with respect to the healthy subjects. They find a strong correlation between the complex geometry and the post-implantations thrombogenicity tendency. In particular large recirculation, regions are strongly related to the geometry modification due to the implantations.

Therefore, it is important to compare post-implantation and migrated Nellix EVAS discussed so far, with the reference cases C and D (healthy and aneurysm conditions), in the context of the previously reported investigations. This discussion is focused on the behavior at the systolic peak and diastolic minimum. In the following, images under analysis are presented from a slightly different view than before, to allow appreciation of the different specific anatomy of each condition.

**Figure 8:**
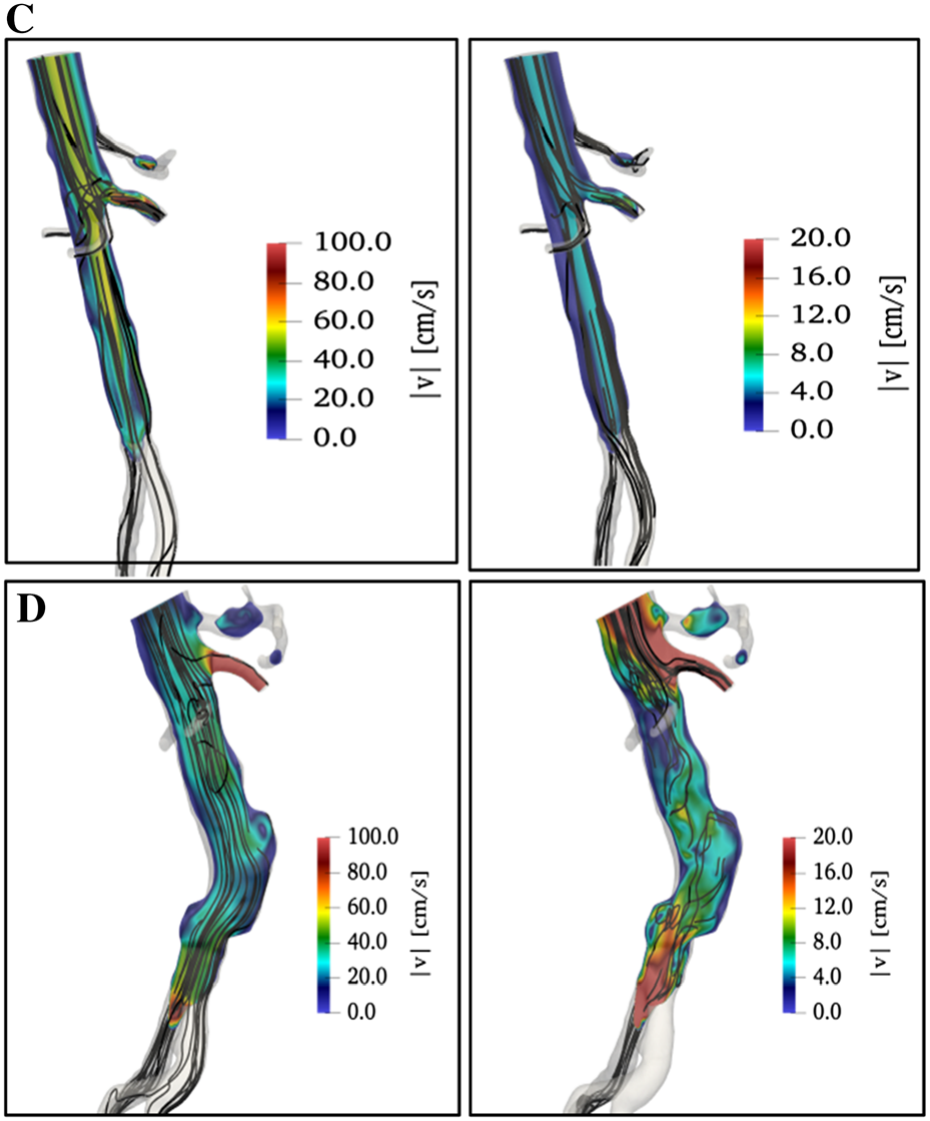
Lateral views of the velocity magnitude (in colors) and of streamlines (black lines) in healthy conditions (configuration C, at the top) and in case of the aneurysm (configuration D, at the bottom). Data at systolic peak (on the left column) and diastolic minimum (on the right). Main flow from top to bottom.

In healthy conditions, refer to the first row of Fig. 8 for the velocity field, no extended recirculation is detected in the rear part of the aorta, both at systolic peak and at diastolic minimum. Streamlines are well aligned both in the bifurcation and in the other lateral branches resulting in a quite homogeneous velocity distribution.

Comparing with this optimal condition, both configurations A and B present substantial criticalities at aorta bifurcation (refer to Fig. 5). Specifically, local changes of velocity are much larger in case A (around 40%) and smaller in case B (around 20%) than in case C, thus confirming qualitatively and extending quantitatively the observations made by Casciaro *et al.*^6^ in their simple numerical model. The overall configuration of the flow field at pressure peak for the Nellix condition after implantation, case A (top left in Fig. 5), is quite similar to healthy case C (top left in Fig. 8), showing a rather efficient hemodynamical state, as shown by Raptis *et al.*^11^

On the other hand, the present study reveals that at pressure minimum this similarity is lost, the Nellix plot showing a much larger level of vortical content (top central plot in Fig. 5) in comparison to the healthy case (top right in Fig. 8), especially at the aorta bifurcation.

This confirms a relationship between the presence of recirculation regions and geometry modification due to implantation, as noted by Tasso *et al.*^14^

In presence of an extended aneurysm, the second row in Fig. 8, several recirculation regions are present, and the streamlines lack the smoothness characteristic of healthy conditions. In all tested conditions, the velocity magnitude is more or less similar, as already noticed in discussing Fig. 5, whereas major differences are observed in velocity spatial and temporal distributions.

**Figure 9:**
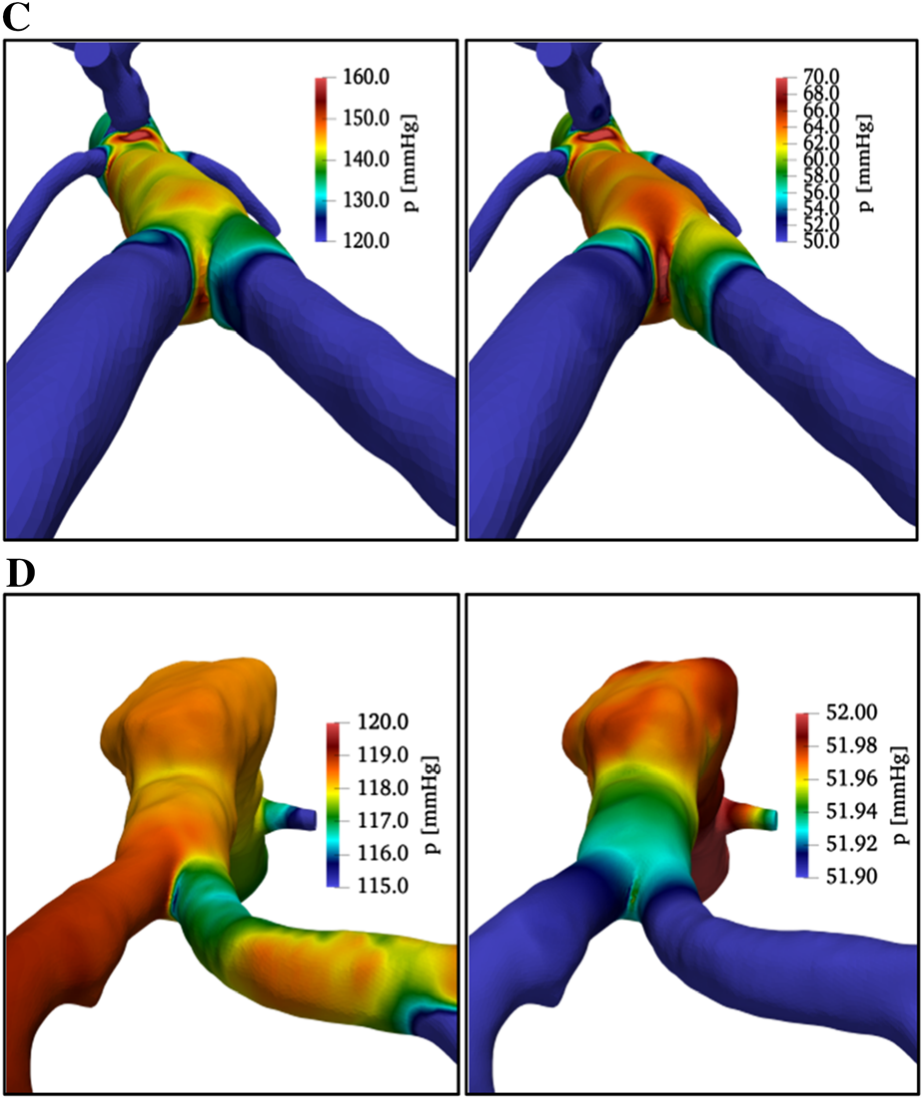
Rear views of wall pressure field (in colors) in healthy conditions (configuration C, at the top) and in case of aneurysm (configuration D, in the middle). Data at systolic peak (on the left column) and diastolic minimum (on the right). Main flow from top to bottom.

Concerning pressure fields, presented in Fig. 9, at first sight, there is a degree of similarity between conditions A and D on one side and B and C on the other.

The aneurysm case D is the only one with asymmetric conditions in the two branches downstream of the bifurcation. However, the detailed inspection of colors and data, as reported in Table 1, reveals more similarity between Nellix migrated configuration (B) and aneurism condition (D), pressure values being different from the other two cases, which are substantially similar.

**Table 1:** Maximum and minimum pressures (in mmHg) before and after aortic bifurcation, for the different tested conditions.

Condition	*p*_min_ (at bifurcation)	*p*_max_ (at bifurcation)	*p*_min_ (after bifurcation)	*p*_max_ (after bifurcation)
A	55	150	55	150
B	60	125	60	120
C	65	145	50	120
D	50	120	50	115

Rather large pressure values are found downstream the bifurcation for the EVAS device when correctly positioned (configuration A), thus indicating a rather small pressure drop in this case. This is in agreement with low pressure drops on AFX, in limbs, and secondary flow in the upper part of the endograft, as reported by Raptis *et al.*^11^,^12^

The previous observation suggests a more careful analysis of pressure variations, especially across the aorta bifurcation. A strong pressure drop is observed across the bifurcation and in all other lateral branches for healthy conditions (around 20 mmHg at the bifurcation, at both systolic peak and minimum pressure). On the contrary, the pressure spatial distribution is much more uniform in all other cases, especially at the systolic peak, and the maximum pressure drop is not larger than a few mmHg (consider in detail the color bars in Figs. 6 and 9). This is indicating a condition in which physiological pressure drops are smoothed by local re-arrangements either in the case of the presence of an aneurysm (case D) and of Nellix EVAS (cases A and B). If this re-arrangement is not taking place within the flow field itself, it is presumable that this would contribute to the overall pressure stress increase on vessel walls.

## Advantages and Limitations

As a matter of fact, the use of high-resolution CFD allows describing specific EVAS geometrical configuration with great care, which is not always possible in an experimental mock-up. Another advantage is that the typical Reynolds numbers are smaller than two thousand (using kinematic viscosity of blood at 35 °C), so that specific turbulence modelling is not required to detail all scales of motion.

Concerning the limitations of the present work, in CFD, the relevant equations are solved on a finite spatial grid using finite time steps. This raises the issue of spatial and temporal resolution. Although in principle the grid can be refined, a limit is posed by the resolution of the CT scan used to generate to flow domain geometry. For the present application, the spatial resolution has been explicitly checked to be of the same order of the smallest length scales in the flow, therefore being sufficient for the description of both the global flow features and of the details of velocity fluctuations.

Concerning time resolution, an inspection of Fig. 4 confirms that time resolution is adequate to describe pressure and velocity variation during heartbeats. A further point to highlight is the dependence of the solution on the boundary conditions used to model inflow and outflow. The main challenge is providing the correct impedance of the downstream part of the system, which is not explicitly solved and may affect the flow behavior in the modeled region. In the literature, the RCR boundary conditions, used in this paper, was proved to be a reliable method, despite small differences induced by the specific settings of the RCR coefficients.^19^ The basic assumption adopted in the present paper is that the impedance at the outflow sections could be modeled by the same set of RCR parameters. For consistency with this assumption, specific attention was paid to select from CT scan approximately the same sections in each outflow branch.

## Conclusions

In this paper, Computational Fluid Dynamics (CFD) is employed as a guide to identifying possible failures of an EVAS system either after implantation and after migration. The key point is the investigation of velocity and pressure fields in order to detect regions with relevant flow recirculation coupled with reduced pressure drops. On the basis of criteria available in the literature, local recirculation induces low values of wall shear stresses which alter endothelial cell mechano-transduction and near-wall transport processes, thus inducing atherosclerosis.^9^ On the other hand, reduced pressure drop conditions are indicators of a possible re-arrangement and increase of pressure values acting on vessel walls.

Both these conditions have been measured in the Nellix EVAS system after lateral migration of the endobags, thus indicating the onset of pathological conditions. This situation is quite close to the very critical one related to the presence of an extended aneurysm. The migration of one or both endobags is supposed to be related to the existing differential pressures acting in the gap formed between the two, which could go on pushing the two branches one away from the other, thus causing aneurysm re-activation and endoleaks.

## References

[1] Ahrens J, Geveci B, Law C (2005). ParaView: An End-User Tool for Large Data Visualization. Visualization Handbook.

[2] Armour CH, Guo B, Pirola S, Saitta S, Liu Y, Dong Z, Xu XY (2020). The influence of inlet velocity profile on predicted flow in type B aortic dissection. Biomech. Model. Mechanobiol..

[3] Ayachit U (2015). The ParaView Guide: A Parallel Visualization Application.

[4] Brownrigg JRW, De Bruin JL, Rossi L, Karthikesalingam A, Patterson B, Holt PJ, Hinchliffe RH, Morgan R, Loftus IM, Thompson MM (2015). Endovascular aneurysm sealing for infrarenal abdominal aortic aneurysms: 30-day outcomes of 105 patients in a single centre. Eur. J. Vasc. Endovasc. Surg..

[5] Carpenter JP, Cuff R, Buckley C, Healey C, Hussain S, Reijnen MMPJ, Trani J, Böckler D, Nellix Investigators (2017). One-year pivotal trial outcomes of the Nellix system for endovascular aneurysm sealing. J. Vasc. Surg..

[6] Casciaro ME, Dottori J, El-Batti S, Alsac JM, Mousseaux E, Larrabide I, Craiem D (2018). Effects on aortoiliac fluid dynamics after endovascular sealing of abdominal aneurysms. Vasc. Endovasc. Surg..

[7] Hang S (2015). TetGen, a delaunay-based quality tetrahedral mesh generator. ACM Trans. Math. Softw..

[8] Lan H, Updegrove A, Wilson NM, Maher GD, Shadden SC, Marsden AL (2018). A re-engineered software interface and workflow for the open-source SimVascular cardiovascular modeling package. J. Biomech. Eng..

[9] Mahmoudi M, Farghadan A, McConnell D, Barker AJ, Wentzel JJ, Budoff MJ, Arzani A (2020). The story of wall shear stress in coronary artery atherosclerosis: biochemical transport and mechanotransduction. J. Biomech. Eng..

[10] Norwood MGA, Lloyd GM, Bown MJ, Fishwick G, London NJ, Sayers RD (2007). Endovascular abdominal aortic aneurysm repair. Postgrad. Med. J..

[11] Raptis A, Xenos M, Kouvelos G, Giannoukas A, Matsagkas M (2018). Haemodynamic performance of AFX and Nellix endografts: a computational fluid dynamics study. Interact Cardiovasc. Thorac. Surg..

[12] Raptis A, Xenos M, Spanos K, Kouvelos G, Giannoukas A, Matsagkas M (2019). Endograft specific haemodynamics after endovascular aneurysm repair: flow characteristics of four stent graft systems. Eur. J. Vasc. Endovasc. Surg..

[13] Rosen RJ, Green RM (2008). Endoleak management following endovascular aneurysm repair. J. Vasc. Interv. Radiol..

[14] Tasso P, Raptis A, Matsagkas M, Rizzini ML, Gallo D, Xenos M, Morbiducci U (2018). Abdominal aortic aneurysm endovascular repair: profiling postimplantation morphometry and hemodynamics with image-based computational fluid dynamics. J. Biomech. Eng..

[15] Thompson MM, Heyligers JM, Hayes PD, Reijnen MMPJ, Böckler D, Schelzig H, De Vries J-PPM, Krievins D, Holden A, Endovascular aneurysm sealing: early and midterm results from the EVAS FORWARD Global Registry (2016). Endovascular aneurysm sealing: early and midterm results from the EVAS FORWARD Global Registry. J. Endovasc. Ther..

[16] Updegrove A, Wilson NM, Merkow J, Lan H, Marsden AL, Shadden SC (2017). SimVascular: an open source pipeline for cardiovascular simulation. Ann. Biomed. Eng..

[17] Van den Ham LH, Holden A, Savlovskis J, Witterbottom A, Ouriel K, Reijnen MM, van den Ham L, Reijnen M, Krievins D, Winterbottom A, Hayes P (2017). Editor’s choice—occurrence and classification of proximal type I endoleaks after endovascular aneurysm sealing using the nellix™ device. Eur. J. Vasc. Endovasc. Surg..

[18] Van Noort K, Overeem SP, van Veen R, Heyligers JMM, Reijnen MMPJ, Schuurmann RCL, Slump CH, Kropman R, de Vries JPPM (2018). Apposition and positioning of the Nellix endovascular aneurysm sealing system in the infrarenal aortic neck. J. Endovasc. Ther..

[19] Vignon-Clementel IE, Figueroa CA, Jansen KE, Taylor CA (2010). Outflow boundary conditions for three-dimensional simulations of non-periodic blood flow and pressure fields in deformable arteries. Comput. Methods Biomech. Biomed. Eng..

[20] Whiting CH, Kenneth EJ (2001). A stabilized finite element method for the incompressible Navier–Stokes equations using a hierarchical basis. Int. J. Numer. Meth. Fluids.

[21] Womersley JR (1955). Method for the calculation of velocity, rate of flow and viscous drag in arteries when the pressure gradient is known. J. Physiol..

[22] Zhou M, Sahni O, Kim HJ, Figueroa CA, Taylor CA, Shephard MS, Jansen KE (2010). Cardiovascular flow simulation at extreme scale. Comput. Mech..

